# Association between peripheral perfusion index and postoperative acute kidney injury in major noncardiac surgery patients receiving continuous vasopressors: a *post hoc* exploratory analysis of the VEGA-1 trial

**DOI:** 10.1016/j.bja.2023.11.054

**Published:** 2024-01-19

**Authors:** Sina Krone, Michael P. Bokoch, Rishi Kothari, Nicholas Fong, Roberta T. Tallarico, Jillene Sturgess-DaPrato, Romain Pirracchio, Alexander Zarbock, Matthieu Legrand

**Affiliations:** 1 Department of Anesthesiology and Perioperative Care, University of California, San Francisco, CA, USA; 2 Department of Anaesthesiology, Intensive Care and Pain Medicine, University Hospital Münster, Münster, Germany; 3 INI-CRCT Network, Nancy, France

**Keywords:** acute kidney injury, haemodynamics, intraoperative hypotension, perioperative outcomes, peripheral perfusion index

## Abstract

**Background:**

The peripheral perfusion index is the ratio of pulsatile to nonpulsatile static blood flow obtained by photoplethysmography and reflects peripheral tissue perfusion. We investigated the association between intraoperative perfusion index and postoperative acute kidney injury in patients undergoing major noncardiac surgery and receiving continuous vasopressor infusions.

**Methods:**

In this exploratory *post hoc* analysis of a pragmatic, cluster-randomised, multicentre trial, we obtained areas and cumulative times under various thresholds of perfusion index and investigated their association with acute kidney injury in multivariable logistic regression analyses. In secondary analyses, we investigated the association of time-weighted average perfusion index with acute kidney injury. The 30-day mortality was a secondary outcome.

**Results:**

Of 2534 cases included, 8.9% developed postoperative acute kidney injury. Areas and cumulative times under a perfusion index of 3% and 2% were associated with an increased risk of acute kidney injury; the strongest association was observed for area under a perfusion index of 1% (adjusted odds ratio [aOR] 1.32, 95% confidence interval [CI] 1.00–1.74, *P*=0.050, per 100%∗min increase). Additionally, time-weighted average perfusion index was associated with acute kidney injury (aOR 0.82, 95% CI 0.74–0.91, *P*<0.001) and 30-day mortality (aOR 0.68, 95% CI 0.49–0.95, *P*=0.024).

**Conclusions:**

Larger areas and longer cumulative times under thresholds of perfusion index and lower time-weighted average perfusion index were associated with postoperative acute kidney injury in patients undergoing major noncardiac surgery and receiving continuous vasopressor infusions.

**Clinical trial registration:**

NCT04789330.

**Editor's key points**

• The perfusion index measured using photoplethysmography is a marker of peripheral tissue perfusion.
• This *post hoc* secondary analysis of a cluster-randomised trial investigated the association between perfusion index and postoperative acute kidney injury in 2534 noncardiac surgery patients, of whom 9% developed acute kidney injury.
• Larger areas and longer cumulative times under thresholds of perfusion index and lower time-weighted average perfusion index were associated with postoperative acute kidney injury.

Intraoperative hypotension is associated with an increased risk of postoperative complications, including acute kidney injury (AKI).^1^, ^2^, ^3^, ^4^ Postoperative AKI is common and associated with longer hospital stay, morbidity, and mortality.^5^^,^^6^ The aetiology of postoperative AKI is complex and multifactorial, but tissue hypoperfusion and hypoxia presumably contribute to the development of AKI.^7^, ^8^, ^9^ It is thus recommended to avoid profound intraoperative hypotension.^10^ The severity and duration of intraoperative hypotension are important determinants of subsequent organ damage.^11^ However, optimal perioperative blood pressure management strategies to reduce complications after noncardiac surgery remain controversial.^10^^,^^11^ Vasopressors are a key intervention to correct anaesthesia-induced vasodilation and hypotension, but in recent years it has also been noted that more liberal use of vasopressors is associated with a higher incidence of AKI.^12^^,^^13^ Haemodynamic management considering variables beyond blood pressure may help prevent postoperative AKI.

The peripheral perfusion index (PI) is defined as the ratio of light absorption between pulsatile and nonpulsatile states obtained through photoplethysmography (Fig. 1). Photoplethysmography is a noninvasive, rapidly available, and commonly used tool for intraoperative patient monitoring. The PI is an indicator of the central and peripheral perfusion status^14^, ^15^, ^16^ and mainly determined by cardiac output and the balance of sympathetic and parasympathetic status that influences vascular tone.^16^ Lower PI values are associated with adverse outcomes in surgical and critically ill patients.^17^, ^18^, ^19^ In a retrospective multicentre cohort study of surgical patients, lower PI values were associated with increased postoperative morbidity and mortality.^20^ Changes in PI after onset of central hypovolaemia may occur earlier than changes in mean arterial pressure (MAP).^21^ The PI may thus provide additional useful information to optimise haemodynamic management.

**Fig 1:**
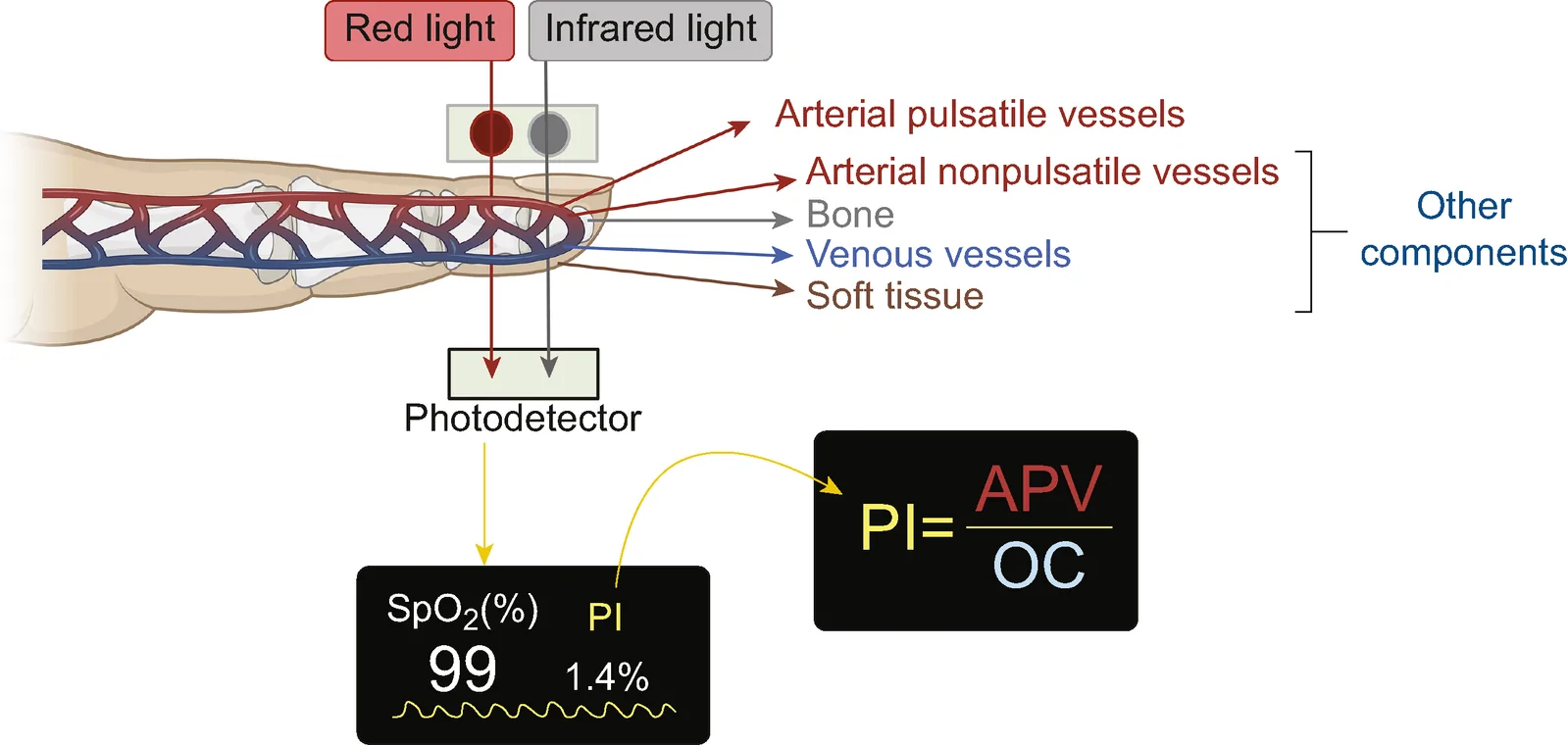
Determinants and calculation of the perfusion index. APV, variation of infrared and red lights absorption related to arterial pulsatile vessels; OC, light absorption of other components, including arterial nonpulsatile vessels, venous vessels, bone, and soft tissue; PI, perfusion index; SpO_2_, oxygen saturation.

In this *post-hoc* exploratory study of a multicentre trial in patients undergoing major noncardiac surgery and requiring continuous infusion of vasopressors, we investigated the association between intraoperative PI and postoperative AKI.

## Methods

### Study design

This was an exploratory *post hoc* analysis of the VEGA-1 trial.^22^ VEGA-1 investigated the feasibility of a pragmatic, multicentre, cluster-randomised trial to compare vasopressors for the treatment of intraoperative hypotension in patients undergoing major surgery (ClinicalTrials.gov Identifier: NCT04789330). VEGA-1 was approved by the Institutional Review Board (University of California, San Francisco, CA, USA), and the requirement for informed consent was waived. This manuscript was written according to STROBE guidelines (see Supplementary material).

VEGA-1 compared phenylephrine with norepinephrine as the first-line i.v. infused vasopressor. Use of a second-line vasopressor and deviation from the randomisation drug were allowed at the discretion of the anaesthesia provider's clinical judgement. It was recommended to maintain MAP within a range of 20% from baseline, but targets were allowed to be adjusted on an individual basis. The primary endpoint for VEGA-1 was compliance with the study protocol; secondary outcomes included AKI and 30-day mortality. The incidence of postoperative outcomes did not differ between patients assigned to norepinephrine or to phenylephrine and we thus included all patients in this secondary analysis.

### Setting

VEGA-1 enrolled patients from five surgical centres of two university hospitals across California (USA). We limited this analysis to four surgical centres owing to availability of PI measurements. Patients were enrolled between July 1, 2021 and December 31, 2021.

### Patients

VEGA-1 included adult patients (≥18 yr old) undergoing major noncardiac surgery under general anaesthesia who received an intraoperative infusion of vasopressors, who had surgery for more than 2 h, and who stayed in the hospital at least one night after surgery. Exclusion criteria included transplant surgery, obstetric procedures, trauma, vasopressor administration before start of the case, transfer to another hospital within 24 h of surgery, and extracorporeal membrane oxygenation.

### Data collection

Data were extracted from the electronic health record, including basic patient characteristics, preoperative and postoperative serum creatinine values, basic intraoperative monitoring, intraoperative vasopressor doses (in norepinephrine equivalents [NEE]^23^ in μg kg^−1^ min^−1^), administered fluids (total crystalloids, albumin, and blood products in ml), and type of anaesthesia, as retrospectively available. Readings for intraoperative monitoring were extracted at 1-min intervals. We included only measurements between the start and end of the surgical procedure to minimise outliers.

PI was obtained using a pulse oximetry probe (Masimo Radical-7; Masimo Corp., Irvine, CA, USA) and the Philips IntelliVue MP70 monitor (Philips North America Corp., Cambridge, MA, USA). Intraoperative PI signals were plotted with other haemodynamic variables retrospectively (Fig. 2), and manually examined for artifacts. After this review, measurements were excluded if PI was equal to 0%, and complete cases were excluded if the standard deviation (SD) of PI measurement series was <0.05% or if >20% of time points (considering 1-min extraction intervals) were missing.

**Fig 2:**
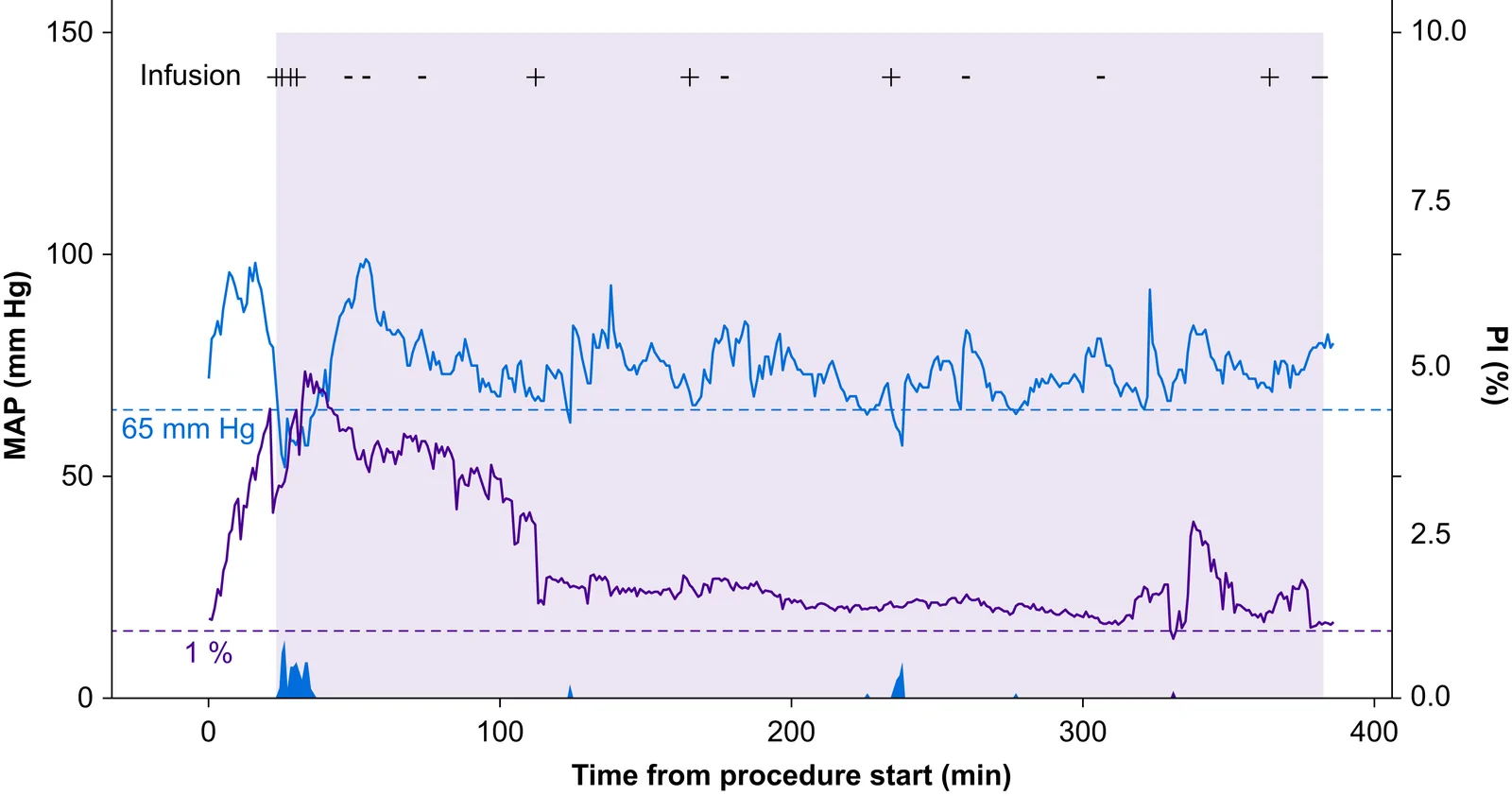
Example plot for measurement of perfusion index (PI) in purple and mean arterial pressure (MAP) in blue during a surgical case. The area under the MAP threshold of 65 mm Hg is presented by the blue area over the abscissa. The shaded area in purple presents a running i.v. vasopressor infusion, increase and decrease shown by the + and – signs.

Blood pressure was measured invasively with arterial catheters or noninvasively with oscillometry. For analyses, one method was selected per patient, with preference given to invasive blood pressure monitoring when available. Blood pressure measurements were considered artifacts and removed if systolic blood pressure was >260 or <40 mm Hg, diastolic blood pressure was >140 or <20 mm Hg, or MAP was >160 or <30 mm Hg.

When calculating time-weighted average blood pressure, we accounted for missing values.^24^ To calculate times under blood pressure thresholds, we linearly imputed missing blood pressure values.

### Outcomes

The primary endpoint was postoperative AKI according to Kidney Disease: Improving Global Outcome (KDIGO) criteria,^25^ considering serum creatinine (≥1.5-fold increase from baseline within 7 days after surgery or 0.3 mg dl^−1^ increase within 48 h), need for renal replacement therapy, or both. Baseline creatinine was defined as the highest value within the 90 days prior to surgery. Severe AKI was defined as stage 2 or stage 3. The 30-day mortality was a secondary outcome.

### Statistical methods

Continuous variables are presented as mean (sd) or median (inter-quartile range [IQR]), as applicable. Categorical variables are presented by count (percentage). Comparisons for categorical data were tested with Pearson's χ^2^ test or Fisher's exact test, continuous variables with independent samples *t*-test or Wilcoxon rank-sum test. Correlations were evaluated using Spearman's correlation coefficient. All associations investigated and corresponding *P*-values should be considered exploratory. Hence, no adjustment was made for false discovery rate. Analyses were performed in R version 4.2.1. (R Foundation for Statistical Computing, Vienna, Austria).

We defined thresholds of PI (1%, 1.5%, 2%, 3%) and MAP (65 mm Hg, 75 mm Hg, 85 mm Hg), and obtained both areas under these thresholds and cumulative times under these thresholds. Areas were calculated with a composite trapezoidal method.^26^ The area under threshold accounts for severity and duration of corresponding events.^11^

To examine the association with AKI, we fitted multivariable logistic regression models. All models were adjusted for covariates we considered clinically relevant, specifically age, sex, comorbidities (preoperative hypertension, diabetes mellitus, congestive heart failure, coronary artery disease), ethnicity, racial identity, randomisation group, surgical service, total volume of intraoperative fluids (crystalloids, albumin, and blood products in ml), and administered vasopressor dose (NEE in μg kg^−1^ min^−1^). Models were assessed for multicollinearity by variance inflation factor. Model fit was evaluated with the Hosmer–Lemeshow test. Sample size was based on retrospectively available data. The variables for area under a PI threshold and area under a MAP threshold were scaled per 100%∗min increase or per 100 mm Hg∗min increase for better interpretability of the odds ratios presented.

As a secondary analysis, we evaluated time-weighted averages for PI and MAP. The time-weighted average is comparable with an arithmetic mean, but accounts for unequal time intervals between measurements and missing values in a time series.^24^^,^^27^

Additionally, we performed sensitivity analyses between patients with invasive and noninvasive blood measurement and investigated the differences in PI among randomisation groups and types of anaesthesia.

## Results

Of 2651 cases who fulfilled the inclusion criteria, 2534 were analysed after exclusion of patients with unreliable PI or MAP measurements (Supplementary Fig. S1). The median age was 63 yr (IQR 51–72), and 1285 patients (51%) were female (Table 1). Invasive blood pressure measurements were available in 1449 cases (57%). After removal of outliers, the mean time between measurements was 2.7 min (1.8 min) for noninvasive and 1.0 min (0.5 min) for invasive blood pressure measurements. The mean percentage of missing PI values (considering 1-min extraction intervals) was 0.7% (min. 0% to max. 20%). Furthermore, 226 patients (9%) developed postoperative AKI, of which 51 (2%) had severe AKI.

**Table 1:** Patient characteristics at inclusion. ACE, angiotensin-converting enzyme; AKI, acute kidney injury; IQR, inter-quartile range; SD, standard deviation; TWA, time-weighted average.

Variable	Overall, *N*=2534	No AKI, *N*=2308	AKI, *N*=226
**Age (yr), median (IQR)**	63 (51–72)	63 (50–72)	67 (56–74)
**Sex, female, *n* (%)**	1285 (51)	1186 (51)	99 (44)
**Acute kidney injury, *n* (%)**			
No AKI	2308 (91)		
Stage 1	175 (6.9)		
Stage 2	39 (1.5)		
Stage 3	12 (0.5)		
**Severe AKI, *n* (%)**	51 (2.0)		
**Preoperative creatinine (mg dl^−1^), mean (****sd****)**	0.88 (0.35)	0.86 (0.31)	1.08 (0.57)
**Postoperative creatinine (mg dl^−1^), mean (****sd****)**	0.93 (0.45)	0.86 (0.30)	1.71 (0.82)
**30-day mortality, *n* (%)**	27 (1.1)	17 (0.7)	10 (4.4)
**Procedure duration (min), median (IQR)**	223 (169–313)	222 (167–311)	245 (182–332)
**Surgical service, *n* (%)**			
Abdominal/Pelvic	752 (30)	644 (28)	108 (48)
Interventional	94 (3.7)	80 (3.5)	14 (6.2)
Neuro	723 (29)	689 (30)	34 (15)
Ortho	625 (25)	581 (25)	44 (19)
Thoracic	85 (3.4)	78 (3.4)	7 (3.1)
Vascular	98 (3.9)	87 (3.8)	11 (4.9)
Other	157 (6.2)	149 (6.5)	8 (3.5)
**Randomisation group, *n* (%)**			
Norepinephrine	1328 (52)	1197 (52)	131 (58)
Phenylephrine	1206 (48)	1111 (48)	95 (42)
**ASA physical status, *n* (%)**			
1	82 (3.2)	80 (3.5)	2 (0.9)
2	1116 (44)	1036 (45)	80 (35)
3	1235 (49)	1,113 (48)	122 (54)
4	98 (3.9)	77 (3.3)	21 (9.3)
5	3 (0.1)	2 (<0.1)	1 (0.4)
**Arterial blood pressure source, *n* (%)**			
Arterial line	1449 (57)	1313 (57)	136 (60)
Noninvasive oscillometric	1085 (43)	995 (43)	90 (40)
**Comorbidities, *n* (%)**			
Hypertension	1091 (43)	976 (42)	115 (51)
Diabetes mellitus	459 (18)	402 (17)	57 (25)
Chronic kidney disease	184 (7.3)	147 (6.4)	37 (16)
Congestive heart failure	116 (4.6)	91 (3.9)	25 (11)
Coronary artery disease	216 (8.5)	195 (8.4)	21 (9.3)
Myocardial infarction	67 (2.6)	61 (2.6)	6 (2.7)
Peripheral vascular disease	89 (3.5)	80 (3.5)	9 (4.0)
Cerebrovascular accident	62 (2.4)	56 (2.4)	6 (2.7)
**Home medication, *n* (%)**			
ACE-inhibitor/angiotensin receptor blocker	752 (30)	674 (29)	78 (35)
Beta-blocker	512 (20)	437 (19)	75 (33)
**Ethnicity, *n* (%)**			
Declined	25 (1.0)	22 (1.0)	3 (1.3)
Hispanic or Latino	414 (16)	372 (16)	42 (19)
Not Hispanic or Latino	2083 (82)	1904 (82)	179 (79)
Unknown	12 (0.5)	10 (0.4)	2 (0.9)
**Patient race, *n* (%)**			
American Indian or Alaska native	44 (1.7)	37 (1.6)	7 (3.1)
Asian	356 (14)	322 (14)	34 (15)
Black or African American	190 (7.5)	176 (7.6)	14 (6.2)
White	1,496 (59)	1,367 (59)	129 (57)
Native Hawaiian or other Pacific Islander	24 (0.9)	21 (0.9)	3 (1.3)
Declined	25 (1.0)	23 (1.0)	2 (0.9)
Other	499 (20)	455 (20)	44 (19)

### Area and time under thresholds of perfusion index and mean arterial pressure

Patients with AKI had larger areas and longer cumulative times under PI and MAP thresholds than patients without AKI (Table 2). The associations between areas and cumulative times under PI and MAP thresholds and AKI are presented in Fig. 3.

**Table 2:** Comparison of area and time under thresholds between acute kidney injury (AKI) groups. Data are presented as median (inter-quartile range). ∗Wilcoxon rank sum test. MAP, mean arterial pressure; PI, perfusion index.

Variable	Overall, *N*=2534^1^	No AKI, *N*=2308^1^	AKI, *N*=226^1^	*P*-value∗
**Area under threshold (PI in %∗min, MAP in mm Hg∗min)**				
**PI <1%**	1 (0–25)	1 (0–24)	3 (0–37)	0.009
**PI <1.5%**	13 (0–90)	12 (0–86)	26 (1–106)	0.008
**PI <2%**	47 (4–171)	45 (3–168)	81 (12–199)	0.002
**PI <3%**	175 (45–372)	169 (41–369)	244 (88–409)	<0.001
**MAP <65 mm Hg**	38 (3–121)	36 (3–116)	63 (7–161)	0.004
**MAP <75 mm Hg**	347 (116–839)	336 (114–826)	580 (155–993)	0.002
**MAP <85 mm Hg**	1443 (702–2634)	1418 (699–2604)	1860 (805–3056)	0.008
**Cumulative time spent below threshold (min)**				
**PI <1%**	6 (0–91)	6 (0–88)	18 (0–110)	0.018
**PI <1.5%**	40 (2–142)	37 (2–140)	70 (6–156)	0.009
**PI <2%**	84 (11–175)	81 (9–172)	115 (34–197)	<0.001
**PI <3%**	144 (67–220)	142 (62–217)	174 (123–251)	<0.001
**MAP <65 mm Hg**	8 (1–24)	8 (1–24)	12 (2–33)	0.004
**MAP <75 mm Hg**	58 (22–122)	57 (22–120)	79 (29–142)	0.006
**MAP <85 mm Hg**	143 (88–223)	142 (88–221)	160 (98–243)	0.051

**Fig 3:**
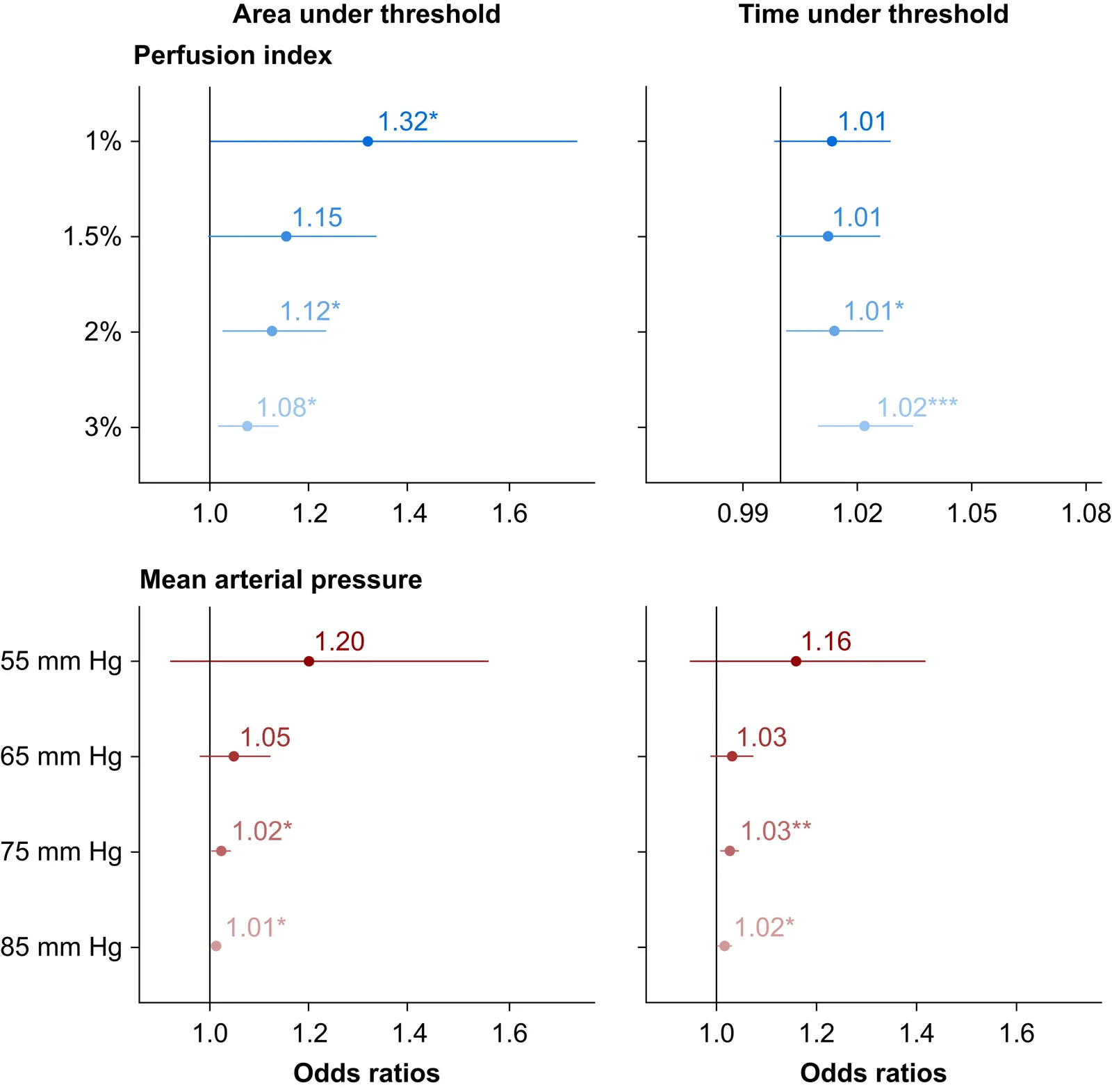
Adjusted multivariable logistic regression models for different thresholds of perfusion index (PI) and mean arterial pressure (MAP) and their association with acute kidney injury. Adjusted odds ratios (aORs) for area under thresholds are scaled per 100 %∗min increase for PI and 100 mm Hg∗min increase for MAP. aORs for time are scaled by 10 min. Dots represent the aOR, lines the 95% confidence interval. Asterisks indicate the *P*-value (∗*P*≤0.05; ∗∗*P*<0.01; ∗∗∗*P*<0.001). Please note the different axis for the plot on time under PI thresholds.

Areas under a PI of 3% and 2% were associated with an increased risk of AKI. The strongest association was observed for area under a PI of 1% (adjusted odds ratio [aOR] 1.32, 95% CI 1.00–1.74, *P*=0.050, per 100 %∗min increase. Cumulative times under PI thresholds of 3% and 2%, scaled per 10 min, were significantly associated with an increased risk of AKI (Fig. 3).

Regarding areas and cumulative times under MAP thresholds, the strongest association with AKI was at 75 mm Hg (aOR 1.02, 95% CI 1.00–1.04, *P*=0.022, per 100 mmHg∗min increase, and aOR 1.03, 95% CI 1.01–1.05, *P*=0.005, per 10 min increase).

### Interaction of perfusion index and mean arterial pressure and the incidence of acute kidney injury

We examined the incidence of AKI stratified by quartiles of time spent with a PI <2% and a MAP <65 mm Hg (Fig. 4). There was a significant difference in the incidence of AKI between groups (*p*<0.001). The incidence of AKI was lowest in patients spending less than 11 min with a PI <2% (Fig. 4). We present this figure with the thresholds 65 mm Hg and 1.5% in Supplementary Figure S2.

**Fig 4:**
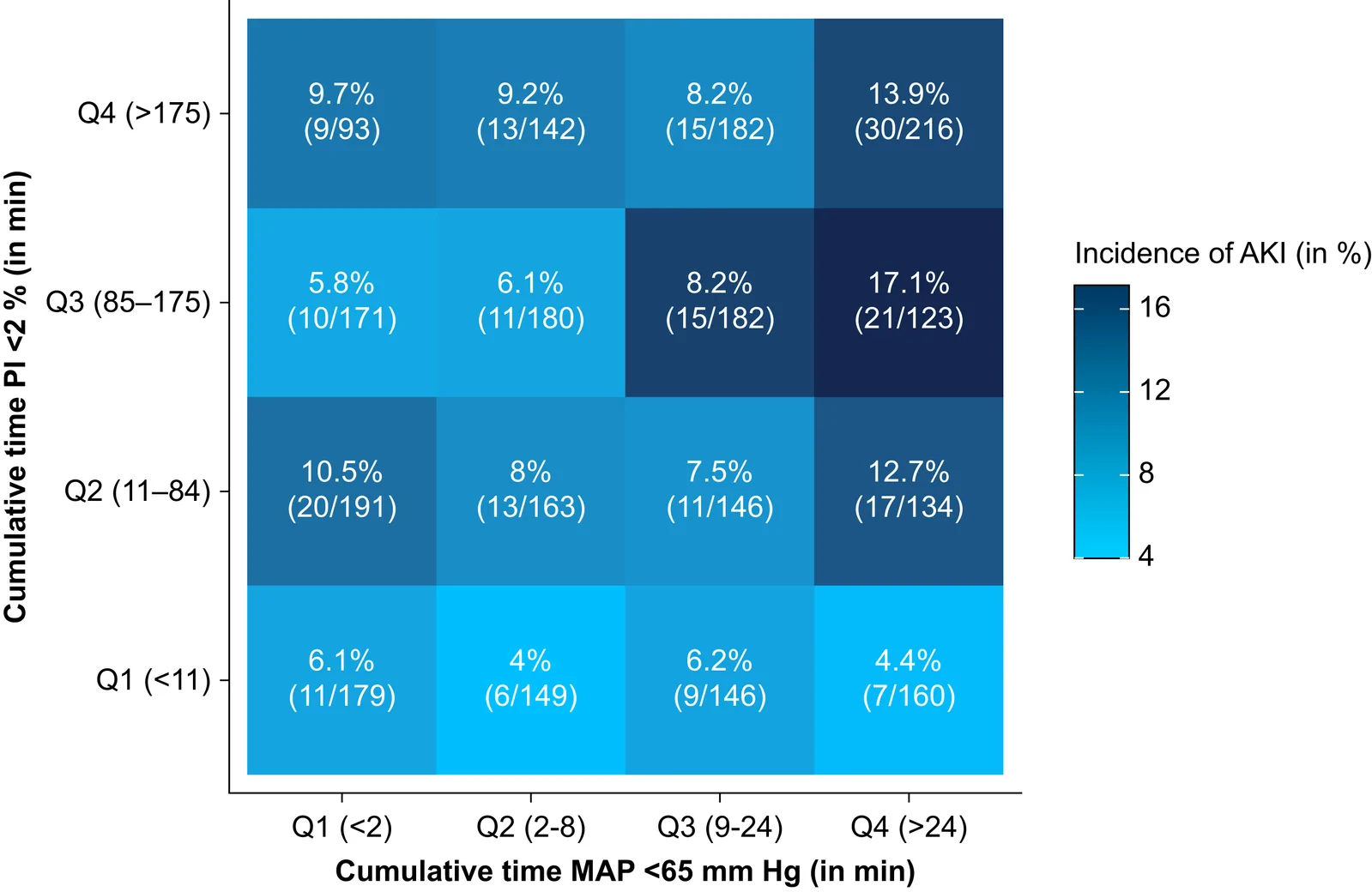
Heatmap of the incidence of acute kidney injury (AKI) by quartiles (Q) of cumulative time spent below thresholds of perfusion index (PI) 1.5% and mean arterial pressure (MAP) 65 mm Hg. This figure illustrates the interaction of PI, MAP, and AKI after combining the patients by quartiles of cumulative time spent with a PI <1.5% and MAP <65 mm Hg. Per combined group, the incidence (new cases/persons at risk) is presented.

### Time-weighted average perfusion index and mean arterial pressure between acute kidney injury groups

Median time-weighted average PI was lower in patients with AKI than in patients without AKI (2.08% [IQR 1.20–3.03] *vs* 2.43% [1.41–3.73], *P*<0.001), whereas there was no difference in time-weighted average MAP (80 mm Hg [75–86] *vs* 81 mm Hg [76–87], *P*=0.160). In multivariable logistic regression analyses, lower time-weighted average PI was associated with AKI (aOR 0.83, 95% CI 0.75–0.92, *P*<0.001), whereas the time-weighted average MAP was not (aOR 0.99, 95% CI 0.97–1.01, *P*=0.264).

### Secondary outcome 30-day mortality

The secondary outcome 30-day mortality was 1%. Patients who died within 30 days of surgery had a lower time-weighted average PI (1.56% [0.68–2.53] *vs* 2.42% [1.40–3.65], *P*=0.002), whereas there was no difference in time-weighted average MAP (81 mm Hg [77–89] *vs* 81 mm Hg [76–87], *P*=0.960). Time-weighted average PI and MAP and cumulative times under PI and MAP thresholds in patients with and without AKI are shown in Supplementary Table S3. Time-weighted average PI was associated with mortality (aOR 0.68, 95% CI 0.49–0.95, *P*=0.024), whereas time-weighted average MAP was not (aOR 1.00, 95% CI 0.95–1.05, *P*=0.957).

### Sensitivity and subgroup analyses

In sensitivity analyses comparing patients with invasive and noninvasive blood pressure monitoring, the association between PI and AKI in logistic regression analyses only remained significant in patients who had invasive monitoring. This remained unchanged when patients who had noninvasive blood pressure were further restricted to patients in whom the blood pressure cuff was documented not to be attached to the side of the pulse oximeter (*n*=546) (Supplementary Tables S1 and S2). Patients who had invasive blood pressure monitoring received higher vasopressor doses (0.05 μg kg^−1^ min^−1^ [IQR 0.03–0.07] *vs* 0.03 μg kg^−1^ min^−1^ [0.02–0.05], *P*<0.001) and spent more time with a MAP <65 mm Hg (11 min [IQR 3–30] *vs* 4 min [0–17], *P*<0.001) than patients who had noninvasive blood pressure monitoring. However, time-weighted average PI did not differ significantly between patients with invasive and noninvasive blood pressure monitoring (2.36% [IQR 1.32–3.64] *vs* 2.46% [1.47–3.69], *P*=0.191).

Vasopressor doses were not relevantly different between patients with and without AKI (0.04 μg kg^−1^ min^−1^ [IQR 0.03–0.06] *vs* 0.04 μg kg^−1^ min^−1^ [0.03–0.07]). The total vasopressor doses administered were only weakly associated with time-weighted average PI (*r*=–0.16) and time-weighted average MAP (*r*=–0.16). However, comparing quartiles of vasopressors administered in NEE in μg kg^−1^ min^−1^ (25th percentile: 0.026; median: 0.040; 75th percentile 0.063), the time-weighted average PI was lower at higher vasopressor doses (Q1: 2.71% [IQR 1.56–4.13]; Q2: 2.56% [1.52–3.77]; Q3: 2.26% [1.39–3.54]; Q4: 2.03% [1.14–3.14]). To further investigate the effect of vasopressor administration on AKI, we fitted the regression models presented in Fig. 3 without adjusting for vasopressor doses (Supplementary Fig. S3).

Median time-weighted average PI was not different between patients who were given phenylephrine or norepinephrine (2.41% [1.42–3.66] *vs* 2.41% [1.35–3.63], *P*=0.509). Information about the type of anaesthesia was available for 1806 patients. There was no difference in median time-weighted average PI between patients with total i.v. anaesthesia (2.47% [IQR 1.44–3.96], *n*=707), inhalational anaesthesia with a minimum alveolar concentration (MAC) <1 (2.46% [1.59–3.71], *n*=632), and a MAC ≥1 (2.49% [1.44–3.83], *n*=467, *P*=0.695). Total median age-adjusted MAC was not correlated with time-weighted average PI (*r*=0.03).

## Discussion

In this *post hoc* exploratory analysis of a pragmatic, multicentre trial, we found that larger areas and longer cumulative times under PI thresholds and lower values of time-weighted average PI are associated with an increased risk for postoperative AKI. Lower time-weighted average PI was associated with 30-day mortality.

Hypotension and hypoperfusion have long been suspected to contribute to postoperative AKI.^7^^,^^8^^,^^10^ Recognising this risk, the use of vasopressors has increased in hospitals in the USA.^12^ Despite the correction and prevention of hypotension, episodes of postoperative AKI still occur. However, data linking intraoperative hypotension to adverse postoperative outcomes (including AKI and mortality) have mostly been collected in observational studies, and a recent meta-analysis suggests that randomised data could not support these findings.^28^ It remains unclear whether and to what extent there is a causal relationship between hypotension and adverse outcomes.^10^^,^^11^

The PI has been studied in various clinical settings, including assessment of regional block success or evaluation of nociception and general anaesthesia.^16^^,^^29^ A small study in 25 healthy adults reported that PI decreases earlier than MAP in response to induced hypovolaemia.^21^

A retrospective multicentre study suggested that a low PI, but not an MAP below 65 mm Hg, is independently associated with severe postoperative complications and 30-day mortality.^20^ Our results differ from this previous retrospective analysis as all of our patients received continuous vasopressors. We also observed that PI was more strongly associated with outcomes compared with MAP. However, blood pressures were closely monitored, and hypotension episodes were rather short and rare in our study,^22^ which could have weakened a potential association between MAP and AKI.

Peripheral alteration of perfusion (using capillary refill time, or peripheral temperature) has been repeatedly associated with organ dysfunction and mortality in critically ill patients receiving vasopressors (mostly sepsis).^30^, ^31^, ^32^ Vasopressor administration could affect PI in various ways. Norepinephrine has predominantly alpha- and beta-adrenoceptor activating properties, whereas phenylephrine is an alpha-adrenoceptor agonist.^33^^,^^34^ Although both vasopressors cause peripheral vasoconstriction, norepinephrine has inotropic properties, which can help maintain or slightly increase cardiac output.^7^ However, we did not observe differences in time-weighted average PI between patients who were given phenylephrine and patients who were given norepinephrine. We observed only a weak correlation between vasopressor doses and time-weighted average PI. Finally, after adjusting for vasopressors doses, we still observed an association between PI and AKI. These results suggest that although vasopressors might have a small impact on PI, vasopressors may not be the main contributor to PI values. Of course, these results should be only considered exploratory, and no causality can be drawn.

Our study was a retrospective exploratory analysis of PI measurements. The primary VEGA-1 trial did not include a protocol for PI measurements. PI data are routinely collected at our institution (even when not displayed on the monitors). Anaesthesia teams were not blinded to PI values (as providers had the possibility to display it on monitors); however, it is highly unlikely that modifications were made based on PI data, as this is not a standard practice in our institution. The quality of the pulse oximetry signal is sensitive to numerous factors (e.g. body or probe positioning, cold extremities).^16^ The incidence of AKI, although consistent with the literature,^35^ is comparatively low. This might be attributed to the selected population receiving vasopressors, resulting in a low incidence of hypotension.^22^

A persistent low PI despite correction of hypotension may be the result of several mechanisms, including excessive vasopressor doses or persistently altered microcirculation unrelated to systemic haemodynamics (e.g. inflammation-related microcirculatory dysfunction or altered baseline microcirculatory function because of chronic cardiovascular disease). The design of our study does not allow differentiating these underlying mechanisms, and further research should focus on investigating factors for altered PI in patients receiving vasopressors.

At our institution, inhaled anaesthesia using sevoflurane is by far the most used general anaesthesia agent for anaesthetic maintenance. Our results might thus not be generalisable to institutions using mostly total i.v. anaesthesia. Of note, sevoflurane has been previously shown to have less impact on PI than other inhaled agents.^36^, ^37^, ^38^ Our sensitivity analysis did not show an association between the dose of inhaled anaesthesia and PI.

### Conclusions

Larger areas and longer cumulative times under thresholds of peripheral perfusion index and lower time-weighted average perfusion index were associated with postoperative acute kidney injury in patients undergoing major noncardiac surgery receiving continuous infusions of vasopressors. Prospective interventional research is needed to investigate if perfusion index-guided intraoperative haemodynamic management can help prevent acute kidney injury.

## Authors’ contributions

Study design: SK, MB, RK, ML

Supervision: ML

Data collection: RK, NF, JS

Figures and tables: SK, RT

Interpretation of results: SK, MB, RK, NF, RT, RP, AZ, ML

Writing of the manuscript: all authors

## Declarations of interest

The authors have no conflicts of interest related to this work. ML reports participating in advisory boards for Spingotec, La Jolla, and Alexion Inc. AZ has received consulting fees from Astute-Biomerieux, Baxter, Bayer, Novartis, Guard Therapeutics, AM Pharma, Paion, Fresenius, research funding from Astute-Biomerieux, Fresenius, Baxter, and speaker's fees from Astute-Biomerieux, Fresenius, Baxter. RP is a consultant for Philips.

## Funding

10.13039/100008984International Anesthesia Research Society IMPACT award and the 10.13039/100000057National Institute of General Medical Sciences of the 10.13039/100000002US National Institutes of Health (T32-GM008440 and R01-GM151494-01).
