# Supplementary material for: Association between peripheral perfusion index and postoperative acute kidney injury in major noncardiac surgery patients receiving continuous vasopressors: a post hoc exploratory analysis of the VEGA-1 trial
Source: Br J Anaesth. 2024 Jan 19;132(4):685–94. doi: 10.1016/j.bja.2023.11.054 (PMC13639447; doi:10.1016/j.bja.2023.11.054)
Supplement: Supplementary file 1 [file mmc1.docx]

I. **SUPPLEMENTARY FIGURES**

**Supplementary Fig S1. STROBE flow chart**
AKI: acute kidney injury; RRT: renal replacement therapy; PI: perfusion index; MAP: mean arterial pressure.

**Supplementary Fig S2. Heatmap of the incidence of AKI by quartiles of cumulative time spent below thresholds of PI 1.5 % and MAP 65 mmHg.**

This figure illustrates the interaction of PI, MAP and AKI after combining the cases by quartiles of cumulative time spent with a PI < 1.5 % and MAP < 65 mmHg. Per combined group, the incidence (new cases/persons at risk) is presented.

Q: quartile; PI: perfusion index; MAP: mean arterial pressure; AKI: acute kidney injury

**Supplementary Fig S3. Adjusted multivariable logistic regression models excluding the covariate vasopressor administration, for different thresholds of PI and MAP and their association with AKI.**

Adjusted Odds Ratios (aOR) for area under thresholds are scaled by 100 %*min for PI and 100 mmHg*min for MAP. aORs for time are scaled by 10 minutes. Dots presenting the aOR, lines the 95% confidence interval. Asterisks presenting the p-value (*p < 0.05, **p < 0.01, ***p < 0.001).

II. **SUPPLEMENTARY TABLES**

**Supplementary Table S1. Multivariable logistic regressions for the association of AKI with perfusion index and mean arterial pressure, separated by the blood pressure measurement source.**

|  | **Invasive BP** | | | **Non-invasive BP** | | |
| --- | --- | --- | --- | --- | --- | --- |
| **Variable** | **aOR^1^** | **95% CI^1^** | **p-value** | **aOR^1^** | **95% CI^1^** | **p-value** |
| **Area under threshold (per 100 %*min for PI, per 100 mmHg*min for MAP)** | | | | | | |
| **PI < 1 %** | 1.44 | 1.05 - 1.96 | 0.022 | 0.90 | 0.46 - 1.72 | 0.748 |
| **PI < 1.5 %** | 1.22 | 1.03 - 1.44 | 0.019 | 0.90 | 0.64 - 1.27 | 0.545 |
| **PI < 2 %** | 1.14 | 1.03 - 1.28 | 0.016 | 0.93 | 0.76 - 1.17 | 0.574 |
| **PI < 3 %** | 1.09 | 1.03 - 1.17 | 0.007 | 0.99 | 0.88 - 1.12 | 0.891 |
| **MAP < 65 mmHg** | 1.05 | 0.97 - 1.14 | 0.202 | 1.06 | 0.92 - 1.22 | 0.398 |
| **MAP < 75 mmHg** | 1.02 | 0.99 - 1.04 | 0.142 | 1.05 | 1.01 - 1.09 | 0.015 |
| **MAP < 85 mmHg** | 1.01 | 0.99 - 1.02 | 0.154 | 1.03 | 1.01- 1.04 | 0.004 |
| **Absolute time spent below threshold (per 10 min)** | | | | | | |
| **PI < 1%** | 1.02 | 1.00 - 1.04 | 0.024 | 0.98 | 0.95 - 1.02 | 0.367 |
| **PI < 1.5 %** | 1.02 | 1.00 - 1.03 | 0.028 | 0.99 | 0.96 - 1.02 | 0.458 |
| **PI < 2 %** | 1.02 | 1.00 - 1.03 | 0.017 | 1.00 | 0.97 - 1.02 | 0.797 |
| **PI < 3 %** | 1.02 | 1.01 - 1.04 | 0.001 | 1.01 | 0.99 - 1.04 | 0.378 |
| **MAP < 65 mmHg** | 1.03 | 0.98 - 1.08 | 0.261 | 1.06 | 0.97 - 1.15 | 0.188 |
| **MAP < 75 mmHg** | 1.02 | 1.00 - 1.04 | 0.136 | 1.05 | 1.02 - 1.09 | 0.002 |
| **MAP < 85 mmHg** | 1.01 | 0.99 - 1.03 | 0.464 | 1.04 | 1.01 - 1.06 | 0.005 |
| ^1^aOR = adjusted Odds Ratio, CI = Confidence Interval BP: blood pressure | | | | | | |

**Supplementary Table S2. Comparison of patient characteristics at inclusion between groups with invasive BP and NIBP measurement.**

| **Variable** | **Invasive BP**,  N = 1,449^1^ | **NIBP**,  N = 1,085^1^ | **p-value**^2^ | |
| --- | --- | --- | --- | --- |
| **Age, years** | 64 (53 - 73) | 61 (48 - 71) | <0.001 | |
| **Gender, female** | 723 (50%) | 562 (52%) | 0.344 | |
| **AKI** |  |  | 0.717 | |
| No AKI | 1,313 (91%) | 995 (92%) |  | |
| Stage 1 | 106 (7.3%) | 69 (6.4%) |  | |
| Stage 2 | 22 (1.5%) | 17 (1.6%) |  | |
| Stage 3 | 8 (0.6%) | 4 (0.4%) |  | |
| **Severe AKI** | 30 (2.1%) | 21 (1.9%) | 0.811 | |
| **Preoperative Creatinine, mg/dL** | 0.89(0.34) | 0.86(0.35) | 0.141 | |
| **Postoperative Creatinine, mg/dL** | 0.94(0.45) | 0.93(0.45) | 0.637 | |
| **30-day mortality** | 22 (1.5%) | 5 (0.5%) | 0.010 | |
| **Procedure duration, min** | 251 (187 - 353) | 194 (151 - 263) | <0.001 | |
| **Surgical service** |  |  | <0.001 | |
| Abdominal/Pelvic | 311 (21%) | 441 (41%) |  | |
| Interventional | 31 (2.1%) | 63 (5.8%) |  | |
| Neuro | 601 (41%) | 122 (11%) |  | |
| Ortho | 273 (19%) | 352 (32%) |  | |
| Other | 74 (5.1%) | 83 (7.6%) |  | |
| Thoracic | 83 (5.7%) | 2 (0.2%) |  | |
| Vascular | 76 (5.2%) | 22 (2.0%) |  | |
| **ASA class** |  |  |  | |
| 1 | 33 (2.3%) | 49 (4.5%) |  | |
| 2 | 528 (36%) | 588 (54%) |  | |
| 3 | 799 (55%) | 436 (40%) |  | |
| 4 | 87 (6.0%) | 11 (1.0%) |  | |
| 5 | 2 (0.1%) | 1 (<0.1%) |  | |
| **TWA MAP** | 81 (76, 86) | 82 (76, 87) | 0.232 | |
| **TWA PI** | 2.36 (1.32 - 3.64) | 2.46 (1.47 - 3.69) | 0.191 | |
| **Randomisation group** |  |  | 0.002 | |
| Norepinephrine | 798 (55%) | 530 (49%) |  | |
| Phenylephrine | 651 (45%) | 555 (51%) |  | |
| **Comorbidities** |  |  |  | |
| Hypertension | 658 (45%) | 433 (40%) | 0.006 | |
| Diabetes | 272 (19%) | 187 (17%) | 0.320 | |
| Chronic kidney disease | 109 (7.5%) | 75 (6.9%) | 0.558 | |
| Coronary artery disease | 142 (9.8%) | 74 (6.8%) | 0.008 | |
| Myocardial infarction | 47 (3.2%) | 20 (1.8%) | 0.030 | |
| Peripheral vascular disease | 59 (4.1%) | 30 (2.8%) | 0.077 | |
| Cerebrovascular accident | 36 (2.5%) | 26 (2.4%) | 0.887 | |
| **Home medication** |  |  |  | |
| ACE inhibitor/Angiotensin receptor blocker | 474 (33%) | 278 (26%) | <0.001 | |
| Beta blocker | 332 (23%) | 180 (17%) | <0.001 | |
| ^1^Median (IQR); n (%); Mean(SD)  ^2^Wilcoxon rank sum test; Pearson's Chi-squared test; Welch Two Sample t-test  AKI: acute kidney injury; TWA: time-weighted average; PI: perfusion index; MAP: mean arterial pressure; BP: blood pressure | | | |  |

**Supplementary Table S3. Comparison of area and absolute times spent below thresholds of PI and MAP between patients deceased and not deceased within 30-days of surgery.**

| **Variable** | **Overall**,  N = 2,534^1^ | **No Death**,  N = 2,507^1^ | **Death**,  N = 27^1^ | **p-value**^2^ | |
| --- | --- | --- | --- | --- | --- |
| **Area under the threshold (PI in % * min, MAP in mmHg * min)** | | | | | |
| **PI < 1%** | 1 (0 - 25) | 1 (0 - 25) | 26 (0 - 78) | 0.007 | |
| **PI < 1.5 %** | 13 (0 - 90) | 12 (0 - 88) | 100 (12 - 177)) | 0.009 | |
| **PI < 2 %** | 47 (4 - 171) | 46 (4 -170) | 178 (41 - 296) | 0.006 | |
| **PI < 3%** | 175 (45 - 372) | 173 (44 - 371) | 352 (136 - 508) | 0.006 | |
| **MAP < 65 mmHg** | 38 (3 - 121) | 38 (3 - 119) | 50 (6 - 227) | 0.200 | |
| **MAP < 75 mmHg** | 347 (116 - 839) | 346 (116 - 836) | 620 (118 - 930) | 0.313 | |
| **MAP < 85 mmHg** | 1,443 (702 - 2,634) | 1,442 (702 - 2,634) | 2,152 (725 - 2,493) | 0.499 | |
| **Cumulative time spent below threshold (in min)** | | | | | |
| **PI < 1%** | 6 (0 - 91) | 6 (0 - 89) | 126 (2 - 179) | 0.009 | |
| **PI < 1.5 %** | 40 (2 - 142) | 39 (2 - 140) | 126 (42 - 212) | 0.018 | |
| **PI < 2 %** | 84 (11 - 175) | 83 (11 - 174) | 157 (64 - 238) | 0.013 | |
| **PI < 3 %** | 144 (67 - 220) | 143 (66 - 220) | 183 (140 - 275) | 0.025 | |
| **MAP < 65 mmHg** | 8 (1 - 24) | 8 (1 - 24) | 11 (4 - 35) | 0.117 | |
| **MAP < 75 mmHg** | 58 (22 - 122) | 58 (22 - 122) | 93 (20 - 113) | 0.569 | |
| **MAP < 85 mmHg** | 143 (88 - 223) | 143 (88 - 223) | 153 (92 - 210) | 0.879 | |
| ^1^Median (IQR) | | | | |  |
| ^2^Wilcoxon rank sum test | | | | |  |
